# Prediction of the Overseas Migration of the Fall Armyworm, *Spodoptera frugiperda*, to Japan

**DOI:** 10.3390/insects14100804

**Published:** 2023-10-06

**Authors:** Akira Otuka

**Affiliations:** Institute for Plant Protection, National Agriculture and Food Research Organization, 2421 Suya, Koshi 8611192, Japan; aotuka@affrc.go.jp

**Keywords:** overseas migration, prediction, GEARN-insect, WRF

## Abstract

**Simple Summary:**

The fall armyworm, Spodoptera frugiperda, is an invasive migratory insect pest that first arrived in Japan in early July 2019. Since then, the species has immigrated to Japan mainly in the summer monsoon season and inflicted damage mainly on the maize used as animal feed in the western region, where major immigrations occur. In this study, to know the precise arrival timing and area of S. frugiperda for the purposes of pest management, a prediction method for its overseas migration from neighboring source areas was developed. The method combines numerical weather predictions and migration calculations. The source areas and take-off and flight behavior of S. frugiperda were input into a migration model that output daily migration prediction figures. A prediction evaluation using 2-year six-point trapping data in Japan showed that the prediction method achieved an average hitting ratio of 78%, indicating the method has sufficient prediction quality for operational use.

**Abstract:**

(1) Background: The fall armyworm, *Spodoptera frugiperda*, is an invasive migratory insect pest that first arrived in Japan in early July 2019. Since then, the species has immigrated to Japan mainly in the summer monsoon season and inflicted damage mainly on the maize used as animal feed in the western region, where major immigrations occur. In this study, to know the precise arrival timing and area of *S. frugiperda* for purposes of pest management, a prediction method for its overseas migration from neighboring source areas was developed. (2) Methods: The method uses the Weather Research and Forecast model to give numerical weather predictions and the GEARN-insect model to predict migration. Emigration source areas on the Chinese mainland and the island of Taiwan and the insect’s take-off and flight behaviors were input to the GEARN-insect model to calculate the daily migration prediction figures. (3) Results: In a prediction evaluation using 2-year six-point trapping data in Japan, the prediction method achieved an average hitting ratio of 78%. (4) Conclusions: The method has sufficient prediction quality for operational use. The technique may be applicable to other migratory moths immigrating to Japan, such as the oriental armyworm, *Mythimna separata*.

## 1. Introduction

Adults of the fall armyworm, *Spodoptera frugiperda* (J. E. Smith), first arrived in East Asia in December 2018 [1]. The invasion, which was estimated to be a migration from Myanmar, was confirmed by light trapping in southwestern Yunnan province, China, and larval progenies were found in January in that province [1]. By May 2019, the insect had already spread to the Yangtze River Valley, with mostly fourth to fifth instar larvae found on maize [2]. Early instar larvae of *S. frugiperda* were also confirmed in southern South Korea in June 2019, and old instar larvae were found in western Japan in early July [3]. This rapid expansion over a wide region illustrates the insect’s strong migratory ability. Migration sources for the first overseas immigrants to Korea and Japan were estimated to be in southern China, which corresponded to the insect’s occurrence there [2,3]. The estimated flight duration of the migrations over the East China Sea to Japan ranged from 18 to 35 h [3], which is longer than the duration of nocturnal migrations over the continent (≤12 or 13 h) [1,3]. The occurrence of *S. frugiperda* in the first season (2019) in Japan was confirmed in 22 prefectures out of 47, mainly in the western region that was the front of the overseas immigration. In the first winter of 2019–2020, *S. frugiperda* continuously populated in the tropical and subtropical regions of southern China, including the islands of Hainan and Taiwan, where the species occurs year-round [4]. As the East Asian summer monsoon began to develop northward in 2020, the population started migrating towards the northern part of the Chinese mainland as well as Japan [5]. Major immigrations to the Japanese mainland started in May 2020, as indicated below, and intermittently continued until the end of the *Bai-u* rainy season (typically mid-July). Since 2020, this regular seasonal migration has consistently occurred. 

*Spodoptera frugiperda* is a polyphagous economic insect species whose host plants include more than 350 plant species [6]. However, the major crop preferred by the East Asian population is maize [5]. Maize cultivation in Japan includes both maize for feed and maize for human consumption (sweet corn). The areas of the former and the latter are 95,500 ha and 21,500 ha, respectively, according to the statistics of the Ministry of Agriculture, Forestry and Fisheries, and the major use of maize in western Japan, where the front of the overseas immigration is located, is for animal feed. Crop protection measures against attacks by migratory insect pests generally constitute migration prediction, occurrence monitoring, and control of the pest at the appropriate time and location. Among these, the migration prediction technique was first developed for rice planthoppers [7]. 

Current prediction methods for the overseas migration of rice planthoppers arriving in Japan use a numerical weather prediction model to predict wind and air temperature and a migration simulation model of Lagrangian type, which calculates the position of migrating planthoppers using forecasted meteorological data [8,9]. Using these methods, a prediction system is operated as an internet-based database service called JPP-NET by the Japan Plant Protection Association [10]. The system generates a migration prediction figure indicating the arrival time and area of rice planthopper immigrants and sends it to plant protection officers. Both migration prediction and light trap monitoring in the field provide basic information for the prediction of the recommended control timing and the area of nymphal stage of the progenies. The same type of protection framework is needed against migratory moth pests. 

Regarding *S. frugiperda*’s migration simulation, several backward and forward trajectory simulations with the insect’s flight parameters of flight speed, heading, temperature ceiling, etc., were conducted to estimate the migration source and destination areas in East Asia [2,3,11,12,13]. A simulation of *Spodoptera frugiperda*’s northward migration in the US was also conducted with a Lagrangian dispersion model combined with biological modelling such as major source areas in spring, degree day development of insect and corn, zone of corn, and its planting date [14,15]. The study simply modelled the insect as an inert particle passively advected by wind, and the results indicated the spatial distribution and mixing of different source populations, with positive relationships between the simulation result and immigration observations [14,15]. Our Lagrangian dispersion model was employed in the current study.

For nocturnal moth species, their flight behavior has been well studied by entomological radar (e.g., Table 13.1 in [16]). It features a take-off at dusk, a subsequent ascent period, favorable flight heights peaking at levels with fast winds or nocturnal temperature inversion (or layer concentrations), faster flight speeds than small insects, orientation and common orientation, landing before dawn over the land (cf. each term in the Index of [16]), and successive nocturnal migrations for three nights (e.g., [3,17]). Implementing these flight properties together with various biological parameters in a migration simulation model can affect the analytical or prediction quality. For example, it is indicated that an insect’s orientation behavior increases its migration distance, changing the destination area [17].

The objective of this study was to develop a method for predicting the migration of *S. frugiperda* over the East China Sea to Japan. The technology for planthopper migration prediction was used as a basis for the development of the method. Among the above flight properties, in the first approximation, this study applied a *S. frugiperda* take-off at dusk, an ascent period, flight speed, temperature ceiling, and a longer flight duration than nighttime (about 10 h) in a novel migration model. Finally, the prediction accuracy of the method was evaluated using recent trapping data in Japan, and the orientation and flight height in relation to the wind structure were discussed.

## 2. Materials and Methods

### 2.1. Predictoin Method

Two numerical models, WRF Version 4.3 and GEARN-insect, were the main components used to predict the overseas migrations of *S. frugiperda* (Supplementary Materials: Figure S1). The Weather Research and Forecast system (WRF) is a non-hydrostatic meso-scale weather prediction model developed by the National Center for Atmospheric Research [18,19] and was used as a numerical prediction model. The WRF is a community model available at https://www.mmm.ucar.edu/models/wrf (accessed on 1 August 2023) that anyone can use for any purpose. The model parameters and weather data used are listed below (Table 1) [20]. 

A WRF forecast run outputs hourly meteorological data, among which, wind, air temperature, and the vertical diffusion coefficient were used by an insect dispersion model, GEARN-insect. The WRF’s original source code was modified to output the vertical diffusion coefficient with a technique in WSPEEDI II Ver. 2.2.0 [21,22,23]. GEARN-insect was originally developed by the former Japan Atomic Energy Research Institute (now the Japan Atomic Energy Agency, Tokai, Japan) in conjunction with the former National Agriculture Research Center (now the National Agriculture and Food Research Organization, Tsukuba, Japan) [8,24,25]. It is a Lagrangian-type particle (insect) dispersion model that makes many insects advect in a downwind direction, and simultaneously diffuse at a rate defined by the diffusion coefficient with a random walk method [24]. The basic equation of motion of a migrating moth with a novel modification for the moth is described as follows [24,26]:

(1) $$x_{t+1}=x_{t}+(u+s_{x})dt$$

(2) $$y_{t+1}=y_{t}+(v+s_{y})dt$$

(3) $$z_{t+1}^{*}=z_{t}^{*}+w^{*}dt+\frac{\partial K_{z}^{*}}{\partial z^{*}}dt+\sqrt{24K_{z}^{*}dt}R+w_{c}^{*}dt$$

valid whereT>13.0 °C

where (*x*, *y*, z*) is the particle position (m). The vertical coordinate is the terrain-following z* coordinate defined by the equations z* = (*z* − *z_g_*)/*h* and *h* = (*z_t_* − *z_g_*)/*z_t_*, where *z*, *z_g_*, *z_t_* are vertical coordinates in a Cartesian coordinate system, the ground height, and the top height of the calculation domain, respectively. (*u*, *v*, w*) is the wind (m/s). The vertical component is in the z* coordinate.

Kz* is the vertical diffusion coefficient (m^2^/s) in the z* coordinate. *Dt* is the time step (s). *R* is a random number from −0.5 to 0.5 [8,17]. (*s_x_*, *s_y_*) is *S. frugiperda*’s horizontal flight speed vector of 3 m/s in the leeward direction (m/s) [3]. The fourth component on the right side of Equation (3) is the random walk factor. The last component *w*_c_* of 0.75 (m/s) was applied to fly upward only during the first 60 min since the initial take-off started. This value was determined based on entomological radar observations such that echoes from insects moved up to typically 1300–1400 m at a maximum of 30 min after sunset (Figure S2) [27], and was calculated as 1350 m/1800 s = 0.75 m/s. *T* is the air temperature, which should be above 13.0 °C [28].

In the model run, the first modelled *S. frugiperda* adult started to take-off at dusk (10:00 UTC) and as many as 500 moths in total continued taking-off for 1 h at a constant temporal rate and randomly in the horizontal position within each southern province in the Chinese mainland (Zhejiang, Fujian, Guangdong, and Jiangxi) and on the island of Taiwan (Figure S3). These areas are possible sources estimated by a previous study [2,3,29]. Moths were assumed to keep flying upward at a speed of 0.75 m/s until 11:00 UTC to capture fast airstreams at altitudes. During their flights, the moths moved leeward at a speed equal to the wind speed plus an insect flight speed of 3.0 m/s [3] (Figure S3). A low limit of air temperature, 13.0 °C [28], was also applied to the moths to keep them from entering the cooler upper air region where the temperature would prevent them from beating their wings. No landing process was implemented in the model due to the lack of knowledge of the process. All the migrating moths from each take-off area were mapped onto a two-dimensional map covering East Asia as open circles of about 18 km in diameter, regardless of their flight heights. The migration arrival of *S. frugiperda* at a trap location in Japan was determined by the passage of any modelled moth (or any circle) within 18 km from the location within 48 h after the prediction started. The forecast time of 48 h was selected because migrations arriving in northern Japan required approximately 48 h in a pre-evaluation. 

### 2.2. Evaluation Method

For prediction evaluation, a hitting ratio was used, which was defined as the ratio of the number of days when the prediction was right (hit) over the number of evaluated days in a prediction period. The period was set as May 2020 (the second season after the 2019 invasion) or May 2021 (the third season), not only because major early migrations started in May [29], but because pheromone trap catches later than May could include both immigrants and locally produced individuals, making the evaluation less accurate. The number of trap locations was six, but one location was replaced during the year due to the end of the survey at a trap location (Izumo to Nagakute in Table 2). These trap sites were distributed from the western to northern parts of Japan.

The determination of whether a prediction failed or not was conducted as follows: when a migration was predicted to arrive at an evaluation trap location on a given date (a positive prediction case) and a corresponding pheromone trap catch was recorded on at least one of 3 or 5 consecutive days starting on the date of predicted arrival, the prediction was right (hit) (Figure S4). The 3-day evaluation period for the positive prediction case was considered to be the time required for an immigrant to arrive at the trap site and to be trapped. A previous analysis of *Spodoptera litura*’s overseas migration suggested that pheromone trapping seemed to have occurred within a 3-day period after airstreams had arrived at the trap site [30]. In the present study, a 5-day period was evaluated as well, in order to see the effect of the longer arrival period on the evaluation. 

When no migration was predicted at a point on a given date (a negative prediction case) and no trap catch was recorded for that date, the prediction was also right (hit). However, evaluation of the negative prediction case was not conducted over the 3- or 5-day period above (Figure S4), as precise evaluation could not be performed due to the possible negative bias in the results by previous immigrants. In the other two cases, i.e., when catches were predicted but did not occur in the 3-day or 5-day evaluation period, and when no catches were predicted but one or more catches occurred, the predictions were considered to have failed. 

The computer used for the prediction was an Intel Xeon E-2288G CPU with a clock speed of 3.70 GHz, 64 GB memory, 500 GB free disk space, and CentOS 8 Stream as the OS.

## 3. Results

**Figure 1:**
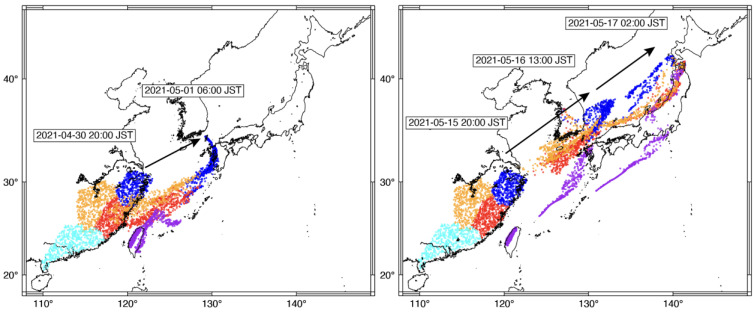
Examples of the prediction starting at 10:00 UTC (19:00 JST) on 30 April 2021 (**left**) and 15 May 2021 (**right**). Colors of the open circles (moths) vary by the take-off area. Later migrating *S. frugiperda* moths from Guangdong province, shown in cyan, were not indicated because they did not contribute to immigration to Japan in the left case, or to a clear depiction of the movement in the right case.

Typical migration prediction figures showed the timing and regions of *S. frugiperda*’s arrival over Japan (Figure 1). For example, in a predicted migration from Zhejiang province, the take-off was at 10:00 UTC on 30 April 2021 and the arrival over western Japan was at 21:00 UTC on the same day, or 06:00 JST on the following day (blue dots in the left panel of Figure 1). Trap catches of 34, 38, and 18 were recorded in the 3-day period (2–4 May 2021) at Koshi (Table 3). Therefore, the prediction for the site was a hit. On the other hand, the prediction at Isahaya was positive too, but no catch was recorded (Table 3). Therefore, the Isahaya prediction failed, suggesting that *S. frugiperda* occurred in a part, not the whole, of Zhejiang province. This migration pattern arriving from Zhejiang province was a typical immigration pattern in western Japan. Weather maps indicated a low-pressure system appearing over the East China Sea on 30 April 2021 and running eastward, generating southwesterly winds over the sea, to the north of Kyushu Island on the following day (top panels of Figure S5).

Another marked example showed a migration in mid-May 2021 travelling over the East China Sea, the Korea Strait, and the Sea of Japan, arriving in both western and northern Japan (right panel in Figure 1; Table S3). Red circles from Fujian province passing over the seas first arrived in Towada, Aomori prefecture, northern Japan, where a trap catch of one insect was correspondingly recorded from 15 to 20 May 2021 (Table S3). The migration distance from Fujian province to Towada was estimated at about 2500 km. A low-pressure system moved northeastward at a speed of about 63.7 km/h on 16 May 2021, crossing over the Yellow Sea, the Korean Peninsula, and the Sea of Japan (lower panels of Figure S5). Daily prediction figures used in the evaluation are available (see Data Availability Statement below).

The average hitting ratios for the two years at the total of 12 trap locations within a 3- or 5-day period were 0.78 and 0.86, respectively (Table 4). 

## 4. Discussion

In this study, a method for predicting the overseas migration of *S. frugiperda* to Japan was developed. The average hitting ratio over 2 years was 78% to 86% depending on the evaluation methods. These values are comparable to the average hitting ratio in the previous migration prediction for rice planthoppers (79%) [8]. The average hitting ratio of the rainfall forecast for the following day in Japan was 82% from May 1992 to 2022 [31]. Therefore, the accuracy of *S. frugiperda*’s migration prediction is also comparable to that of the rainfall forecast.

The hitting ratio differed both among locations and years. The hitting ratio for Isahaya in 2021 was very low, which was caused by many prediction failures in early May (Table 3). Many immigrants were caught in the same period at Koshi, a trap site to the southeast of Isahaya; thus, it was suggested that no *S. frugiperda* adults within the source area linked to the migration to Isahaya at that time could have occurred circumstantially.

A slight increase in the hitting ratio occurred in different evaluation periods (Table 4), producing more positive hits. This result suggests that male moth immigrants might exhibit female-searching behavior for at least 5 days after their arrival at the destination. Such an extended trapping behavior is quite different from the action of a net trap or suction trap for rice planthoppers, which directly capture flying immigrants and mostly produce instant catch peaks at their arrival [32]. This difference is a reason for why longer temporal periods (3–5 days) were required to evaluate the prediction method for the moth, while a daily evaluation was sufficient in the previous study on rice planthoppers. 

Although the *S. frugiperda* migration prediction model showed a sufficient prediction quality for operational use, there may be room for improvement. Entomological radar studies have shown that high-flying insects actively select fast, high-altitude airstreams moving in a direction that is highly beneficial for their seasonal migration [17,33], and that the insects adopt optimal flight headings to partially compensate for the cross wind drift, maximizing travelling distances [17]. Among the various flight properties, the current model did not consider *S. frugiperda*’s orientation property nor select fast wind levels for simplicity. The former could affect the predicted arrival time and area at the destination [17]. The orientation of *S. frugiperda* and/or *Helicoverpa zea* (Boddie) observed by a radar in June 1985 to 1990 in southern Texas, US, has been reported. The insect’s crab angle was found to be about +89° or +60° when the wind was southeasterly or southwesterly, respectively [34]. The crab angle is the angle between the wind and body alignment directions, which ranges from −90° (counterclockwise) to +90° (clockwise); it is 0° when the two directions are parallel. The result suggested that these moths adjust their flight directions to the northeast, which is like the flights of *Autographa gamma* and other high-flying insects in the UK [17]. *Spodoptera frugiperda*’s crab angle over the Chinese mainland was assumed to be +30° in previous trajectory analyses [2,11,12,13]. The crab angle of the migration over the East China Sea is unknown due to lack of observations there. Instead, a simulation approach to investigate the relationship between the crab angle of various values and the hitting ratio of the migration prediction for Japan is a good next step to improve the prediction quality.

In addition, this study fixed the take-off time over a wide source region for simplicity, which might have caused prediction errors. For improvement, the take-off time should be set for each province at least in the next version of GEARN-insect.

Insect flight height selection is also an important flight property affecting the prediction quality. High-flying nocturnal moths over a flat landmass favorably select a nocturnal temperature inversion top level of typically 100–400 m above ground level (AGL) (e.g., Figure 10.8 in [16]) or the level with the fastest wind in the atmospheric boundary layer (e.g., Figure 10.10 in [16]). These phenomena can often be observed at night with a clear sky when the ground surface is cooled by radiation and the wind near the surface is not very strong. Radar observations of moths migrating in a weak low-level jet at 100–200 m AGL have been reported when a trough approached a radar site in southeastern Australia [35]. 

However, the wind system assisting *S. frugiperda*’s overseas migration to Japan is typically a cyclone-induced low-level jet in the warm and humid southern sector of the *Baiu* front ([36], Figure S5). This jet is stronger than that in the above Australian case. Southwesterly winds of 10–20 m/s over the sea typically blow at 1000–1500 m above sea level (ASL) (Figure 4a in [37]). However, the direction of similar strong winds at lower or upper levels gradually changes to southerly or westerly, respectively (Ekman spiral) (Figure 4a in [37]). The same wind structures are confirmed by radiosonde data from Fukuoka Meteorological Station in northern Kyushu Island, where two low-pressure systems responsible for *S. frugiperda* immigration in Japan passed to the north of the city (Figure S5, Tables S4 and S5). As the air temperature at 1500 m ASL is typically about 16–17 °C (the same as a rice planthopper’s temperature ceiling) or higher in the *Baiu* rainy season (Figure 6 in [25]), moths tolerant to a relatively lower air temperature may be able to fly at a slightly higher level. However, since the insect’s possible sources are located to the southwest of western Japan [2,3], flights at the level of southwesterly winds are favorable for the migration to Japan. As illustrated with black arrows in Figure 1, the predicted migrations run towards the northeast, indicating many simulated moths flew at the above migration-favorable level. Hence, the results suggest that the ascent movement after take-off, the temperature ceiling, and the vertical diffusion, which determine the insect’s flight height, are the key to distributing the moths at various height levels when they emigrate and migrate over the sea. In fact, it was previously highlighted that *S. frugiperda* migrants arriving in Japan might have had various flight heights from 500 to over 1000 m ASL based on the relationship between the trap locations at the destination and the topology in the upwind immigration direction, assuming over-the-mountain flights [3].

The initial goal of the development of the migration prediction method was to predict the correct timing for the chemical control of descendent larvae based on predicted dates and areas. Although a prediction method has already been successfully developed, the number of *S. frugiperda* immigrants arriving in Japan was found to be very low in recent years (Tables S2 and S3). So far, damage caused by the larvae of the next generation after this immigration to the spring maize seeding in April in western Japan has not been severe, and a prediction system is not always necessary in such field situations. Rather, the moth often causes damage to the summer maize seeding in July and August, when its density is higher than in the spring crop season. 

Nonetheless, this new technique could be applicable to other migratory moth species, such as the oriental armyworm *Mythimna separata*. The first generation of *M. separata* generally arrives in Japan in late May to early June [38]. An intense mass immigration can occasionally occur and cause serious damage to feed maize, pastures, and rice [38]. The outbreak of *M. separata* in the early summer of 2017 in western Japan is such an example [39,40,41,42]. No one noticed the initial immigration in each prefecture, and descendent old-instar larvae had already caused severe damage to crops by the time people finally noticed them [43]. Unfortunately, it was too late to control them at that point [43]. The prediction of such mass immigration is necessary both to provide advance notification of the migration and to achieve effective control of larvae at their young instar stage. 

This simulation approach could also be applicable to other land regions such as China or North America [14,15] and other weather conditions like the nocturnal temperature inversion in early summer or autumn [44,45]. Note that various model parameters, such as orientation, layering, flight speed, etc., should be properly modelled to fit the target insect’s flight behavior in each migration case. 

This study, therefore, presents the scientific basis and a method for predicting the overseas migration of a moth species.

## Figures and Tables

**Table 1:** WRF parameters, meteorological data, and mapping software for the prediction.

Parameter/Data	Values
*WRF parameters*:	
domain size	151, 131
number of domains	1
horizontal resolution (*dx*, *dy*)	27 km
map projection	Mercator
projection reference latitude	32.0 degree
projection reference longitude	128.0 degree
forecast time	72 h
time step	120 s
microphysics	Thompson scheme
cumulus parameterization	Tiedtke scheme
longwave radiation	RRTMG scheme
shortwave radiation	RRTMG shortwave
planetary boundary layer	Mellor-Yamada-Janjic scheme
surface layer	Eta similarity
land surface	Noah Land Surface Model
analysis nudging	applied
*Meteorological data*:	
name	NOAA Global Forecast System (GFS)
time interval	6 h
horizontal resolution	0.25 degree
number of vertical layers	23 layers up to p_top
pressure top (p_top)	50 hPa
URL for downloading	https://registry.opendata.aws/noaa-gfs-bdp-pds/ (accessed on 1 August 2023)
*Mapping software*:	
name	The General Mapping Tools Ver. 6.3.0

**Table 2:** Location of pheromone traps used for prediction evaluation in 2020 and 2021.

2020	2021
Minami-satsuma (31.48° N, 130.34° E)	Minami-satsuma (31.48° N, 130.34° E)
Koshi (32.88° N, 130.74° E)	Koshi (32.88° N, 130.74° E)
Isahaya (32.83° N, 130.02° E)	Isahaya (32.83° N, 130.02° E)
Nangoku (33.59° N, 133.64° E)	Nangoku (33.59° N, 133.64° E)
Izumo (35.33° N, 132.75° E)	Nagakute (35.16° N, 137.08° E)
Towada (40.62° N, 141.16° E)	Towada (40.62° N, 141.16° E)

**Table 3:** Example of trap catch and migration prediction at two trap sites in western Japan in 2021.

Date of Collection/ Prediction	Trap Location: Koshi					Isahaya				
	Prediction ^1^ (Source)	FT ^2^ (h)	Trap Catch	Eval. 3-Day ^3^	Eval. 5-Day ^3^	Prediction (Source)	FT (h)	Trap Catch ^4^	Eval. 3-Day	Eval. 5-Day
1 May 2021	Yes (ZJ)	12	0	H	H	Yes (ZJ)	11	0	F	F
2 May 2021	No		34	-	-	No			-	-
3 May 2021	No		38	-	-	No			-	-
4 May 2021	Yes (JX)	38	18	H	H	Yes (GD)	35		F	F
5 May 2021	No		9	-	-	No		0	-	-
6 May 2021	Yes (TW)	35	17	H	H	Yes (TW)	33		F	F
7 May 2021	Yes (ZJ)	29	22	H	H	Yes (ZJ)	24	0	F	F
8 May 2021	Yes (ZJ)	23	3	H	H	Yes (ZJ)	21		F	F
9 May 2021	Yes (ZJ)	37	9	H	H	Yes (ZJ)	32		F	F
10 May 2021	Yes (FJ)	36	13	H	H	Yes (FJ)	34		F	F
11 May 2021	No		0	-	-	No			-	-
12 May 2021	Yes (JX)	35	2	H	H	Yes (ZJ)	26	0	F	H
13 May 2021	Yes (FJ)	29	3	H	H	Yes (FJ)	27		F	H
14 May 2021	Yes (FJ)	32	1	F	H	Yes (FJ)	29		H	H
15 May 2021	Yes (ZJ)	20	0	F	H	Yes (ZJ)	17		H	H
16 May 2021	Yes (TW)	20	0	H	H	Yes (TW)	18		H	H
17 May 2021	No		0	-	-	No		7	-	-
18 May 2021	No		0	-	-	No		0	-	-
19 May 2021	Yes (ZJ)	30	1	H	H	Yes (FJ)	31	0	H	H
20 May 2021	Yes (TW)	20	0	H	H	Yes (GD)	32	2	H	H
21 May 2021	No		0	-	-	No		19	-	-
22 May 2021	No		3	-	-	No		3	-	-
23 May 2021	Yes (TW)	36	0	H	H	Yes (ZJ)	36	2	H	H
24 May 2021	No		1	-	-	No		3	-	-
25 May 2021	No		0	-	-	No		7	-	-
26 May 2021	Yes (ZJ)	11	0	F	F	Yes (TW)	30		H	H
27 May 2021	No		0	-	-	No		2	-	-
28 May 2021	Yes (ZJ)	23	0	H	H	Yes (ZJ)	20	0	H	H
29 May 2021	No		0	-	-	No		3	-	-
30 May 2021	No		0	-	-	No		0	-	-
31 May 2021	No		1	F	-	No			F	-

^1^ Yes indicates that migration was predicted; No means no migration was predicted. ^2^ FT: flight time from the initial take-off time; ^3^ H: the prediction was a hit; F: the prediction failed. ^4^ The collection was performed daily or at 2- to 5-day intervals (gray cells).

**Table 4:** Hitting ratio of *S. frugiperda*’s overseas migration prediction.

Location	Hitting Ratio	
	3-Day Period	5-Day Period
2020:		
Minami-satsuma	0.67	0.88
Koshi	0.76	0.93
Isahaya	0.89	0.89
Nangoku	0.64	0.81
Izumo	0.83	0.76
Towada	0.96	0.95
**Yearly avg.**	**0.79**	**0.87**
2021:		
Minami-satsuma	0.83	1.00
Koshi	0.78	0.94
Isahaya	0.44	0.59
Nangoku	0.79	0.87
Izumo	0.84	0.75
Towada	0.91	0.95
**Yearly avg.**	**0.77**	**0.85**
**Total avg.**	**0.78**	**0.86**

## Data Availability

Daily prediction figures in May 2020 and 2021 are available at https://jppn.sakura.ne.jp/faw/prediction/2020 (accessed on 1 August 2023) and https://jppn.sakura.ne.jp/faw/prediction/2021 (accessed on 1 August 2023), respectively.

## Supplementary Materials

The following supporting information can be downloaded at https://www.mdpi.com/article/10.3390/insects14100804/s1: Figure S1, Components of the migration prediction method; Figure S2, Target number versus flight height of a typical moth obtained by the entomological radar; Figure S3, Schematic of migration modelling; Figure S4, Evaluation method; Figure S5, Surface weather map at 00:00 UTC; Table S1, Specifications of NARO’s entomological radar [46,47]; Table S2, Migration prediction and its evaluation in 2020; and Table S3, Migration prediction and its evaluation in 2021. Table S4, Surface and upper data at Fukuoka at 00:00 UTC, 1 May 2021; Table S5, Surface and upper data at Fukuoka at 12:00 UTC, 16 May 2021.
