# Supplementary material for: Prediction of the Overseas Migration of the Fall Armyworm, Spodoptera frugiperda, to Japan
Source: Insects. 2023 Oct 6;14(10):804. doi: 10.3390/insects14100804 (PMC10607009; doi:10.3390/insects14100804)
Supplement: Supplementary file 1 [file insects-14-00804-s001.zip › insects-2597493-supplementary.pdf]

Supplementary Materials

This document presents supplementary figures and tables for a paper entitled "**Prediction of the overseas migration of the fall armyworm, *Spodoptera frugiperda*, to Japan**"

Schematic of the method

The prediction method consists of two numerical models and a mapping tool (Figure S1).

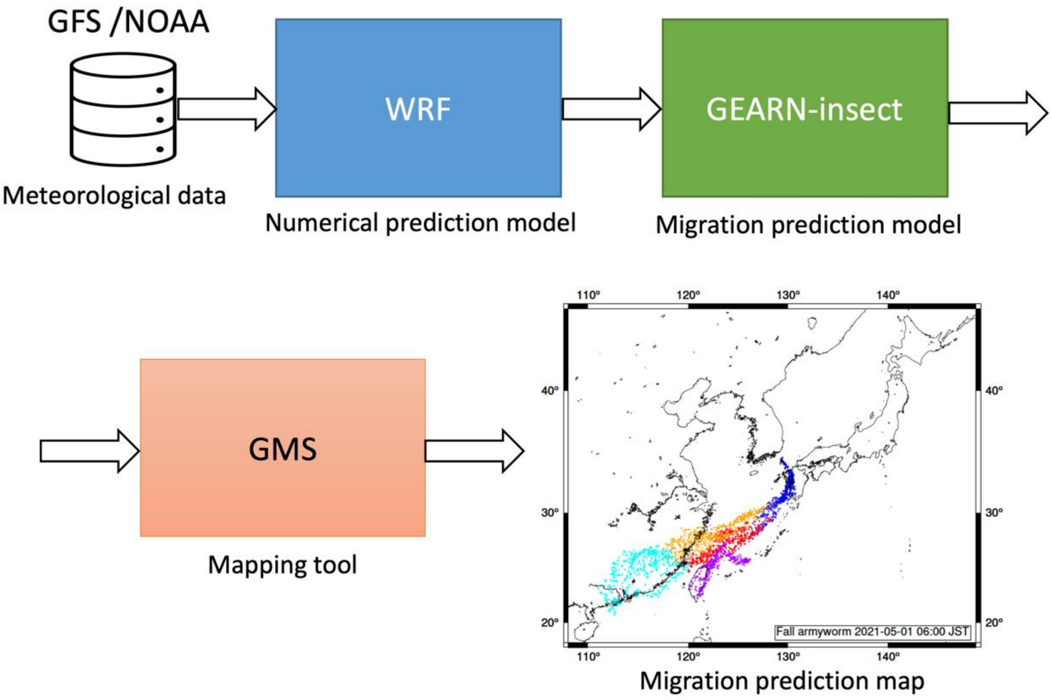

Figure S1 Components of the migration prediction method.

Estimation of the upward velocity of moths at dusk in Japan

The study used a post-take-off ascent velocity of 0.75 m/s for *Spodoptera frugiperda*. The value was estimated as follows. An X-band entomological radar of vertical-looking-radar type with the specifications indicated in Table S1 was operated in Saga prefecture, western Japan in October 2016, when the aerial density of the flying moths was high. *Spodoptera litura*-like target echoes with an estimated radar cross section a_0 of 0.4 to 1 cm^2 were selected [46]. Their species were not identified. The radar cross section a_0 is related to the moth weight (p. 64–65 in [16]). The moth target number before and after sunset indicated that many moths took off soon after sunset and reached to a maximum height level of 1,400 m above the ground within 30 min (Figure S2). This observation indicated that the maximum ascent velocity was 0.75 m/s (=1,350 m / 1,800 sec). The ascent velocity at 900 m on the orange line was 0.47 m/s (=850 m / 1,800 sec). The values of 1,350 m and 850 m are the center of the 1,400-m and 900-m height intervals, respectively.

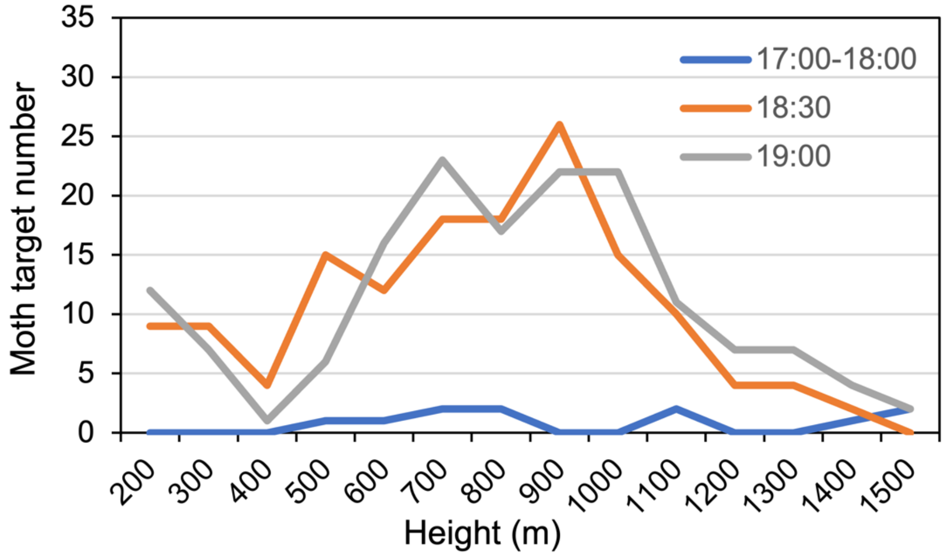

Figure S2 Target number versus flight height of typical moths observed by the entomological radar. An ascent movement just after take-off at dusk in Japan is shown. The location was at a point (33.31°N, 130.33°E) in Saga city, Saga Prefecture, western Japan. The observation date was 6 October 2016, and sunset occurred at 17:57 Japan Standard Time on this date.

Table S1 Specifications of NARO's entomological radar

Parameter	Value
**Radar type**	Vertical looking radar in narrow-angle conical scan with a rotating double dipole feed [47]
**Wavelength**	3.2 cm (microwave of 9.41 GHz)
**Polarization**	horizontal linear polarization
**Pulse width**	70 ns
**Pulse repetition**	2250 Hz
**Peak power**	25 kW
**Parabolic antenna**	1.5 m in diameter
**Antenna gain**	41 dB
**Analysis method**	Time domain method by Hammer and Drake [47]

Migration modelling

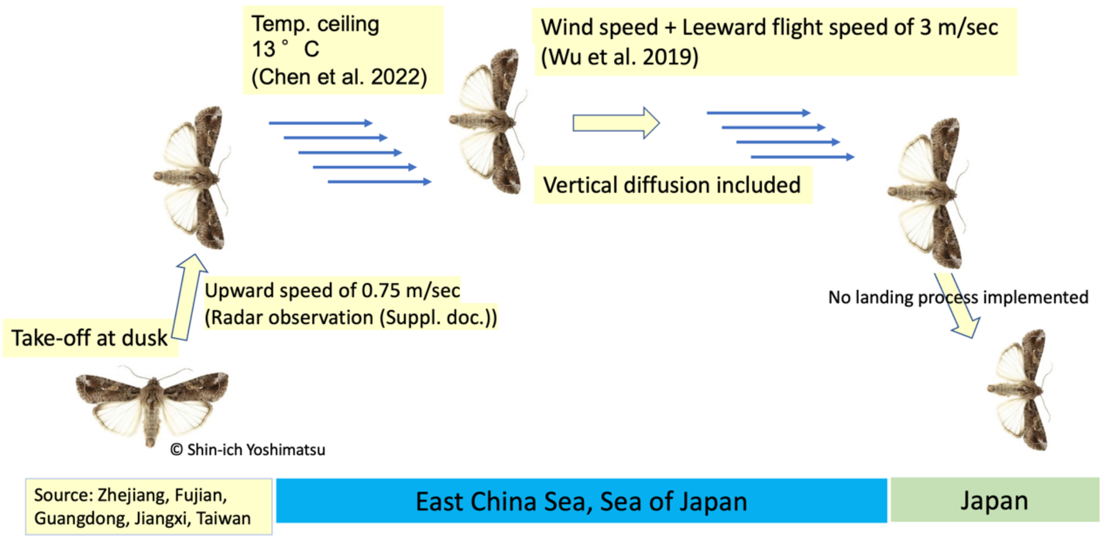

Figure S3 Schematic of migration modelling.

Evaluation method

Date of collection / prediction	Trap catch	Prediction	Flight time (h)	Evaluation by 3-day period	Evaluation by 5-day period
Day 0	0	Yes	12	H	H
Day 1	1	No		-	-
Day 2	2	No		-	-
Day 3	0	No		H	-
Day 4	0	No		H	-
Day 5	0	No		F	F
Day 6	1	Yes	32	F	H
Day 7	0	No		-	-
Day 8	0	No		-	-
Day 9	0	No		F	-
Day 10	1	No		H	-
Day 11	0	No			

Figure S4 Evaluation method. Moth samples in the trap were collected in the morning and the catch number was recorded on that date. A positive prediction (Yes) was evaluated with trap catches over a 3-day period (blue) or 5-day period (green). A negative prediction (No) was evaluated with a catch on the following date (black or brown). H: the prediction was hit; F: the prediction failed. Some negative predictions after a positive prediction (-) were not evaluated to avoid a biased evaluation by possible lingering catches. A shorter flight time from the initial take-off indicates a faster immigration with stronger winds.

Weather maps

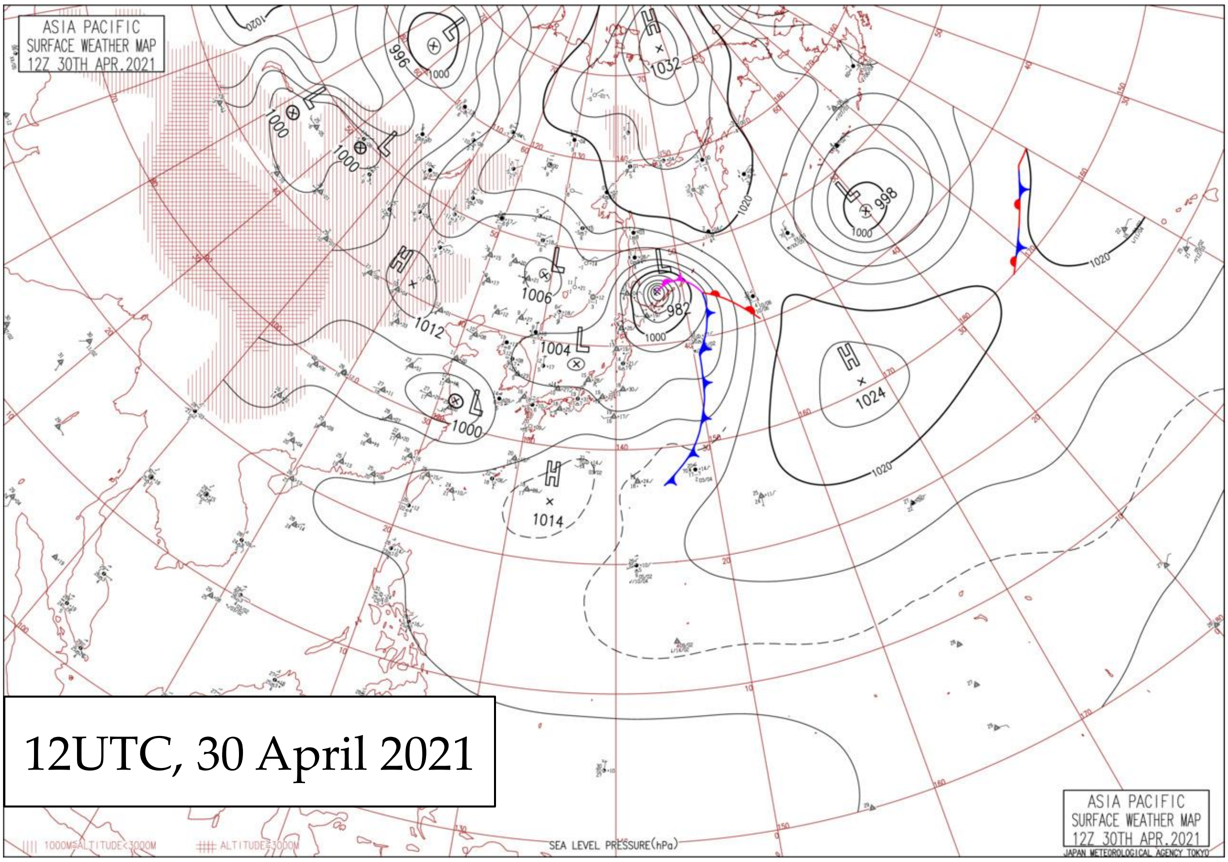

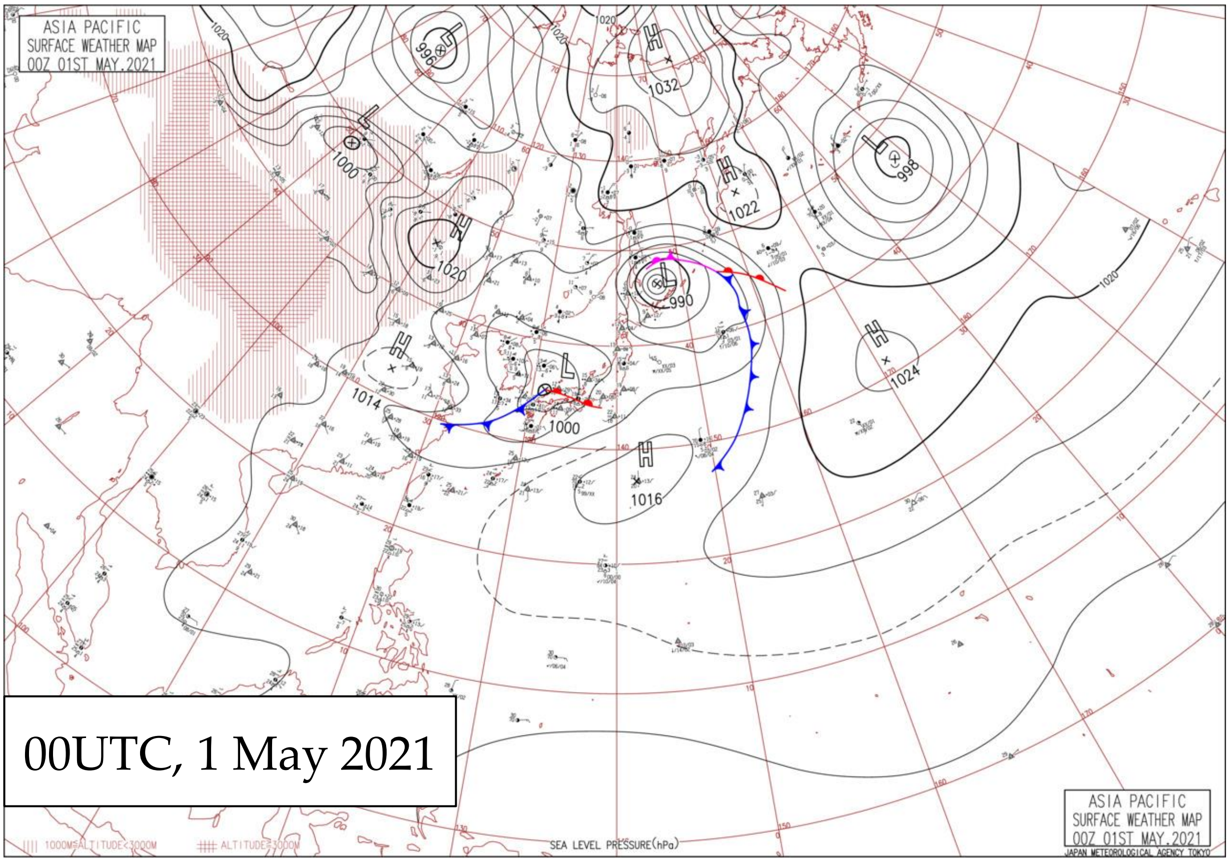

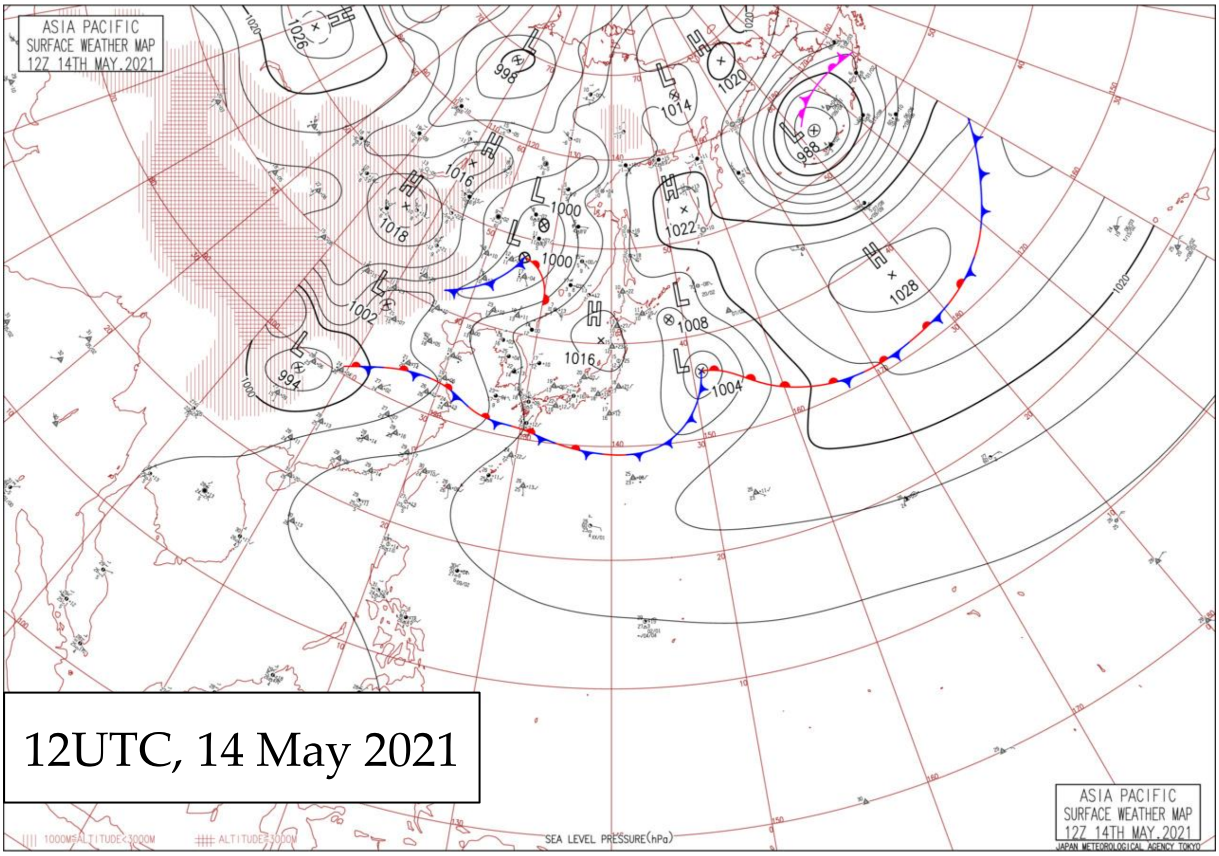

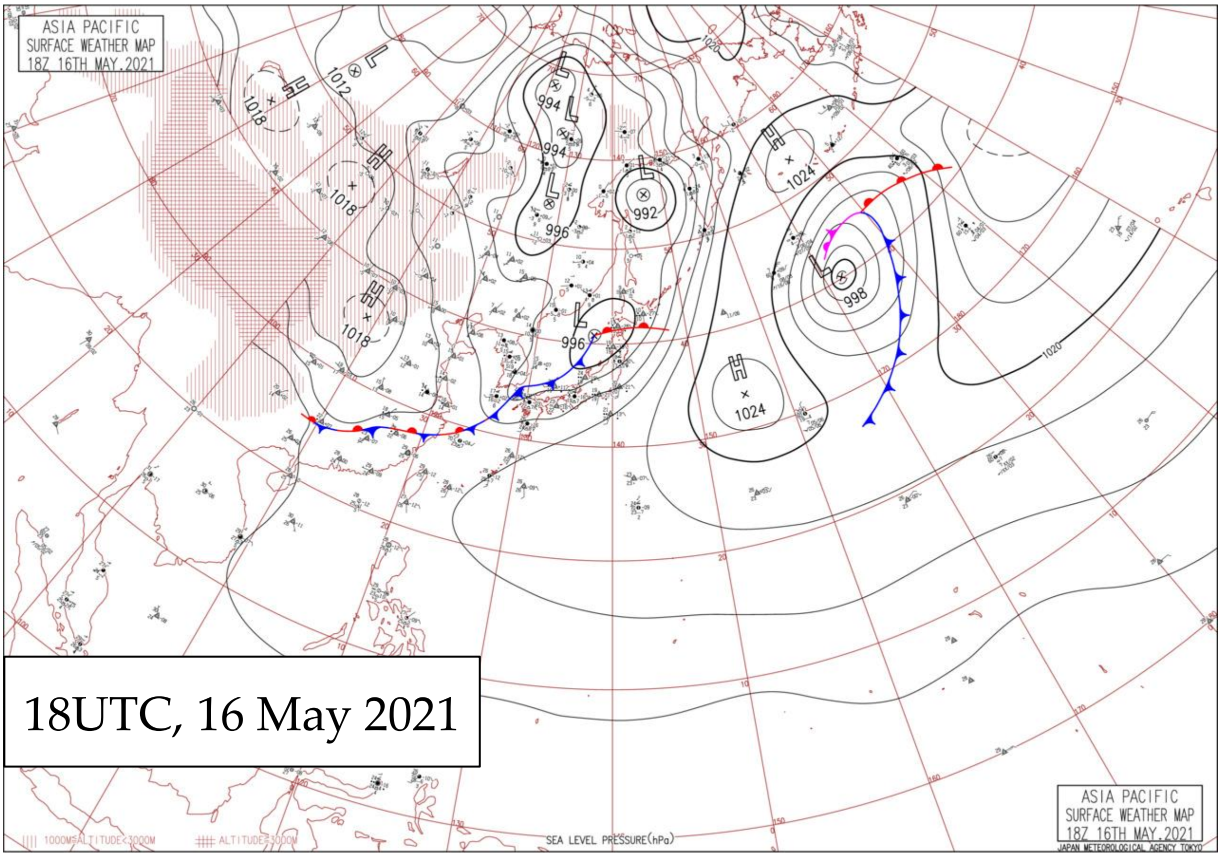

Figure S5 Surface weather maps related to emigration (upper) and immigration (lower) of the two migration events arriving in Japan on 1 and 16 May 2021. The maps were made by the Japan Meteorological Agency.

Table S2 Migration prediction and its evaluation in 2020

Date of collection / prediction	Location: Minami-satsuma					Koshi					Isahaya					Nangoku					Izumo					Towada				
	Prediction (Source)	FT (h)	Trap catch	Eval. 3-day	Eval. 5-day	Prediction (Source)	FT (h)	Trap catch	Eval. 3-day	Eval. 5-day	Prediction (Source)	FT (h)	Trap catch	Eval. 3-day	Eval. 5-day	Prediction (Source)	FT (h)	Trap catch	Eval. 3-day	Eval. 5-day	Prediction (Source)	FT (h)	Trap catch	Eval. 3-day	Eval. 5-day	Prediction (Source)	FT (h)	Trap catch	Eval. 3-day	Eval. 5-day
01/05/2020	No		0	H	H	No		0	H	H	No			-	-	No		0	H	H	No		Setting	H	H					
02/05/2020	No		0	H	H	No		0	H	H	No			-	-	No		0	H	H	No			H	H					
03/05/2020	Yes (FJ)	24	0	F	F	Yes (ZJ)	29	0	F	H	Yes (ZJ)	24		H	H	Yes (TW)	32		F	H	Yes (ZJ)	35		F	F					
04/05/2020	No		0	-	-	No		0	-	-	No			-	-	No		0	-	-	No			-	-					
05/05/2020	No		0	-	-	No		0	-	-	No			-	-	No			-	-	No			-	-			Setting		
06/05/2020	No		0	H	-	No		0	F	-	No			-	-	No		0	H	-	No			H	-	No			H	H
07/05/2020	No		0	H	-	No		1	H	-	No		1	-	-	No			F	-	No			H	-	No			H	H
08/05/2020	No		0	H	H	No		0	H	H	No			-	-	No		1	H	H	No		0	H	H	No		0	H	H
09/05/2020	Yes (FJ)	23	0	F	H	Yes (FJ)	20	0	H	H	Yes (FJ)	20		H	H	Yes (GD)	32	0	H	H	Yes (JX)	27		F	F	No			H	H
10/05/2020	Yes (FJ)	23	0	F	H	No		0	-	-	No			-	-	No		0	-	-	No			-	-	Yes (JX)	46		F	F
11/05/2020	No		0	-	-	No		0	-	-	No			-	-	No		0	-	-	No			-	-	No			-	-
12/05/2020	Yes (ZJ)	44	0	H	H	No		1	H	-	No			-	-	No		2	F	-	No			H	-	No			-	-
13/05/2020	No		0	-	-	No		0	H	-	No			-	-	No		1	H	-	No			H	-	No		0	H	-
14/05/2020	No		1	-	-	No		0	H	H	No			-	-	No		0	H	H	No			H	H	No			H	-
15/05/2020	Yes (TW)	26	1	F	H	Yes (TW)	23	0	H	H	Yes (GD)	31		H	H	Yes (TW)	32	0	F	F	Yes (JX)	30		F	F	No			H	H
16/05/2020	Yes (GD)	26	0	F	H	Yes (FJ)	20	0	H	H	Yes (FJ)	17		H	H	Yes (FJ)	23		F	H	No			-	-	No			H	H
17/05/2020	Yes (ZJ)	24	0	H	H	Yes (ZJ)	26	0	H	H	Yes (ZJ)	24		H	H	No		0	-	H	No			-	-	No			H	H
18/05/2020	Yes (FJ)	25	0	H	H	Yes (FJ)	27	1	H	H	Yes (FJ)	25	1	H	H	Yes (FJ)	35		H	H	Yes (FJ)	34		F	F	No		0	H	H
19/05/2020	No		0	-	-	No		3	-	-	No			-	-	No		0	-	-	No			-	-	No			H	H
20/05/2020	No		3	-	-	No		3	-	-	No			-	-	No		0	-	-	No			-	-	No			H	H
21/05/2020	No		0	F	-	No		2	F	-	No			-	-	No		1	F	-	No		0	H	-	No			H	H
22/05/2020	No		1	H	-	No		4	F	-	No			-	-	No		1	H	-	No			H	-	No			H	H
23/05/2020	No		0	H	H	No		4	F	F	No			-	-	No			H	H	No			H	H	No			H	H
24/05/2020	No		0	F	F	No		3	H	H	Yes (ZJ)	37	1	H	H	No		0	H	H	No			H	H	No		0	H	H
25/05/2020	Yes (ZJ)	46	1	H	H	Yes (ZJ)	40	0	H	H	No			-	-	No			F	F	No			H	H	No			H	H
26/05/2020	No		0	-	-	No		1	-	-	No			-	-	No		1	H	H	No			H	H	No			H	H
27/05/2020	No		1	-	-	No		2	-	-	No			-	-	No		0	F	F	No			H	H	No			H	H
28/05/2020	Yes (ZJ)	28	1	H	H	Yes (ZJ)	26	1	H	H	Yes (ZJ)	23	2	H	H	Yes (ZJ)	33	1	H	H	No			H	H	No			H	H
29/05/2020	No		0			No		0	-	-	No			-	-	No			-	-	No		0	H	H	No		0	H	H
30/05/2020	Yes (TW)	35	0	H	H	No		0	-	-	No			-	-	No		0	-	-	No			H	H	No			H	H
31/05/2020	Yes (ZJ)	25	1	H	H	Yes (ZJ)	33	1	H	H	Yes (ZJ)	33		F	F	No		0	H	-	No			H	H	No			H	H
Total catch			10					27					5					8					0							

Table S3 Migration prediction and its evaluation in 2021

Date of collection / prediction	Minami-satsuma					Koshi					Isahaya					Nangoku					Nagakute					Towada				
	Prediction (Source)	FT (h)	Trap catch	Eval. 3-day	Eval. 5-day	Prediction (Source)	FT (h)	Trap catch	Eval. 3-day	Eval. 5-day	Prediction (Source)	FT (h)	Trap catch	Eval. 3-day	Eval. 5-day	Prediction (Source)	FT (h)	Trap catch	Eval. 3-day	Eval. 5-day	Prediction (Source)	FT (h)	Trap catch	Eval. 3-day	Eval. 5-day	Prediction (Source)	FT (h)	Trap catch	Eval. 3-day	Eval. 5-day
01/05/2021	Yes (ZJ)	11	0	H	H	Yes (ZJ)	12	0	H	H	Yes (ZJ)	11		F	F	Yes (ZJ)	17		F	F	Yes (JX)	24	0	F	F	No		Setting	H	H
02/05/2021	No		1	-	-	No		34	-	-	No		0	-	-	No		0	-	-	No			-	-	No			H	H
03/05/2021	No		0	-	-	No		38	-	-	No			-	-	No			-	-	No			-	-	No			H	H
04/05/2021	Yes (GD)	41	0	H	H	Yes (JX)	38	18	H	H	Yes (GD)	35		F	F	No			H	-	No		0	H	-	No		0	H	H
05/05/2021	Yes (ZJ)	17	0	H	H	No		9	-	-	No		0	-	-	No		0	H	-	No			H	-	No			H	H
06/05/2021	Yes (TW)	38	0	H	H	Yes (TW)	35	17	H	H	Yes (TW)	33		F	F	No			H	H	No			H	H	No			H	H
07/05/2021	Yes (ZJ)	20	1	H	H	Yes (ZJ)	29	22	H	H	Yes (ZJ)	24		F	F	No		0	H	H	No			H	H	No			H	H
08/05/2021	Yes (ZJ)	26	1	H	H	Yes (ZJ)	23	3	H	H	Yes (ZJ)	21		F	F	Yes (ZJ)	31		F	F	Yes (ZJ)	37		F	F	Yes (ZJ)	42	0	F	F
09/05/2021	Yes (ZJ)	26	0	H	H	Yes (ZJ)	37	9	H	H	Yes (ZJ)	32	0	F	F	No		0	-	-	No		0	-	-	No			-	-
10/05/2021	Yes (TW)	33	0	H	H	Yes (FJ)	36	13	H	H	Yes (FJ)	34		F	F	No			-	-	No			-	-	No			-	-
11/05/2021	Yes (TW)	26	1	H	H	No		0	-	-	No			-	-	No		0	H	-	No			H	-	No			H	-
12/05/2021	Yes (FJ)	20	1	H	H	Yes (JX)	35	2	H	H	Yes (ZJ)	26		F	H	No		0	H	-	No			H	-	No		0	H	-
13/05/2021	Yes (FJ)	23	1	H	H	Yes (FJ)	29	3	H	H	Yes (FJ)	27		F	H	No		0	H	H	No			H	H	No			H	H
14/05/2021	Yes (FJ)	25	0	H	H	Yes (FJ)	32	1	F	H	Yes (FJ)	29	0	H	H	No			H	H	No		0	H	H	No			-	-
15/05/2021	Yes (TW)	25	2	H	H	Yes (ZJ)	20	0	F	H	Yes (ZJ)	17		H	H	Yes (FJ)	28		F	H	No			H	H	No			-	-
16/05/2021	Yes (TW)	23	1	H	H	Yes (TW)	20	0	H	H	Yes (TW)	18		H	H	Yes (TW)	24	0	F	H	Yes (TW)	29		F	F	Yes (FJ)	31		H	H
17/05/2021	Yes (ZJ)	17	0	H	H	No		0	-	-	No		7	-	-	Yes (ZJ)	17		H	H	No			-	-	No		1	-	-
18/05/2021	Yes (ZJ)	20	2	F	H	No		0	-	-	No		0	-	-	No			-	-	No			-	-	No			-	-
19/05/2021	Yes (FJ)	23	0	F	H	Yes (ZJ)	30	1	H	H	Yes (FJ)	31	0	H	H	Yes (TW)	35	0	H	H	No		0	H	-	No			F	-
20/05/2021	Yes (TW)	20	0	H	H	Yes (TW)	20	0	H	H	Yes (GD)	32	2	H	H	Yes (TW)	24		H	H	No			H	-	No			H	-
21/05/2021	No		0	-	-	No		0	-	-	No		19	-	-	No			-	-	Yes (GD)	47		H	H	No			H	H
22/05/2021	No		0	-	-	No		3	-	-	No		3	-	-	No		2	-	-	No			-	-	No			H	H
23/05/2021	Yes (ZJ)	30	1	H	H	Yes (TW)	36	0	H	H	Yes (ZJ)	36	2	H	H	No			-	-	No			-	-	No		0	H	H
24/05/2021	Yes (ZJ)	17	0	H	H	No		1	-	-	No		3	-	-	Yes (TW)	41		H	H	Yes (TW)	48	1	H	H	No			H	H
25/05/2021	No		2	-	-	No		0	-	-	No		7	-	-	No			-	-	No			-	-	No			H	H
26/05/2021	Yes (ZJ)	12	3	F	H	Yes (ZJ)	11	0	F	F	Yes (TW)	30		H	H	Yes (TW)	36	0	H	H	No			-	-	No			H	H
27/05/2021	No		0	-	-	No		0	-	-	No		2	-	-	Yes (ZJ)	17	0	H	H	Yes (ZJ)	27		H	H	No			H	H
28/05/2021	Yes (ZJ)	23	0	H	H	Yes (ZJ)	23	0	H	H	Yes (ZJ)	20	0	H	H	Yes (ZJ)	28		H	H	Yes (ZJ)	34		H	H	No		0	H	H
29/05/2021	No		0	-	-	No		0	-	-	No		3	-	-	No			-	-	No		1	-	-	Yes (ZJ)	42		H	H
30/05/2021	No		2	-	-	No		0	-	-	No		0	-	-	No		4	-	-	No			-	-	No			-	-
31/05/2021	No		1	F	-	No		1	F	-	No			F	-	No			-	-	No			H	-	No		1	-	-
Total catch			20					175					48					6					2					1		

Table S4 Surface and upper data at Fukuoka at 00:00 UTC, 1 May 2021

Surface data

Pressure (hPa)	Altitude (m)	Temp. (℃)	Relative humidity (%)	Wind speed (m/s)	Wind direction (°)
1001.2	18	19.5	67	4.2	260

Upper data

Pressure (hPa)	Geopotential height(m)	Temp. (℃)	Relative humidity (%)	Wind speed (m/s)	Wind direction (°)
1000	28	18.5	70	5	265
925	689	12.9	79	15	246
900	920	11.4	73	17	254
850	1395	8.1	72	23	258
800	1895	8.8	15	28	251
700	2979	-0.9	32	30	259

Data from the Japan Meteorological Agency

Table S5 Surface and upper data at Fukuoka at 12:00 UTC, 16 May 2021

Surface data

Pressure (hPa)	Altitude (m)	Temp. (℃)	Relative humidity (%)	Wind speed (m/s)	Wind direction (°)
1002.3	18	27.4	75	5.7	200

Upper data

Pressure (hPa)	Geopotential height(m)	Temp. (℃)	Relative humidity (%)	Wind speed (m/s)	Wind direction (°)
1000	38	27.1	75	6	224
925	723	21.6	91	19	211
900	961	20.1	96	23	221
850	1454	17.9	96	25	230
800	1973	15.2	98	26	235
700	3095	9.6	95	32	242

Data from the Japan Meteorological Agency
